# Oral Cancer in HSCT Pediatric Patients Arising on GVHD: A Comprehensive Review

**DOI:** 10.3390/cancers14235775

**Published:** 2022-11-24

**Authors:** Tiziana Cantile, Noemi Coppola, Federica Canfora, Daniela Adamo, Elvira Ruoppo, Michele Davide Mignogna, Stefania Leuci

**Affiliations:** 1 Oral Medicine Unit, Department of Neuroscience, Reproductive and Odontostomatological Sciences, University of Naples Federico II, 80131 Naples, Italy; 2 Department of Medicine, Surgery and Dentistry, Scuola Medica Salernitana, 84121 Salerno, Italy

**Keywords:** pediatric oncology, hematopoietic stem cell transplantation, HSCT, GVHD, OSCC, oral cancer, secondary malignancies, pediatrics

## Abstract

**Simple Summary:**

This brief review describes cases of oral cancer arising from GVHD in patients who underwent haematopoeitic stem cell transplantation (HSCT) as children. The risk of developing secondary solid malignancies after HSCT is well known, and, of the various cancers, oral squamous cell carcinoma (OSCC) is one of the most common. In the literature, there are no summary studies on this topic in the population of patients who have been transplanted as children. So, here we report all the published data on HSCT pediatric patients, affected by oral GVHD, who have been developed OSCC, with the aim of promoting the prevention of oral cancer in this patient setting.

**Abstract:**

After haematopoietic stem cell transplantation and a history of GVHD, the risk of developing secondary malignancies, including oral cancer, is higher. This risk increases with time post-transplantation; therefore, pediatric patients undergoing HSCT, who have long-term survival chances, are in a high-risk category. The aim of this review is to provide data on HSCT, GVHD, clinical manifestations, histological features and treatment of oral cancer, and outcomes in HSCT pediatric patients, affected by oral GVHD, who have been developed OSCC. Descriptive statistics were used to validate data. Fifteen studies on a total of 33 patients were selected. Data on oral cancer showed that the tongue was the most frequently involved site (13 pts; 39.39%), followed by the floor of the mouth (4 pts; 12.12%), and buccal mucosa (4 pts; 12.12%). Oral squamous cell carcinoma was the histological feature reported. There were 19 (57.58%) deaths occurring between 2 and 46.5 months after OC diagnosis. Eleven patients survived with a median follow-up of 34 months. Considering the high risk of developing oral cancer, a conventional oral examination every 6 months is recommended for HSCT pediatric patients who have developed GVHD.

## 1. Introduction

Hematopoietic stem cell transplantation (HSCT) represents an effective procedure, widely used as a therapeutic treatment for patients diagnosed with various malignant haematological disorders, bone marrow failure disease, and congenital immune deficiencies [1].

Yearly, more than 50,000 HSCT are performed worldwide, of which more than 21,000 are allogeneic [2].

Stem cells may be obtained from different sources: mobilized peripheral blood (PB), bone marrow (BM), and umbilical cord blood (UCB) [3]. The selection of a donor is a critical element contributing to the HSCT’s success. Cells may be obtained from the patient (autologous, HLA identical); an identical twin (syngeneic, HLA identical); a sibling, relative, or unrelated donor (allogeneic, which can be HLA identical, haploidentical, or mismatched) [4].

Improvements in transplant medicine have considerably increased the patient’s long-term survival rate: 70–80% of those who survive the first 2 years following HSCT are expected to become long-term survivors [5]. With the improvement in long-term survival rate, there is a need to quantify the late effects of HSCT [6].

In addition to a broad range of complications, such as infections, cardiovascular impairment, end-stage renal disease, bronchiolitis obliterans, and avascular necrosis, the development of secondary malignancies (hematologic malignancies, lymphoproliferative disorders, and solid secondary cancers) has been reported [7]. The risk of developing secondary malignancies reaches its peak among children who have undergone transplantation when they are 10 years old, and it is higher for those who are 10 to 29 years old at the time of transplantation than for those who are 30 years or older [8].

Hematological malignancies and lymphoproliferative diseases are relatively common and usually present early after the HSCT [6]; solid cancers (involving the skin, oral cavity, genitourinary tract, breast, bone, connective tissue, brain, liver, lung, and thyroid) are less frequent with an incidence rate of 2 to 6% at 10–15 years after HSCT and account for approximately 5% to 10% of late death in patients after HSCT [1,2]. The risk of secondary solid tumors begins to increase around 10 years after transplantation and continues even 20 years later [1]. In fact, the cumulative incidence of secondary solid malignancies is 2.5% (95% CI: 2.0–3.0%) at 10 years, 5.8% (95% CI: 4.3–7.0%) at 15 years, and 8.8% (95% CI: 6.2–12.3%) at 20 years after HSCT, respectively [2].

In particular, the oral cavity is thought to be one of the most susceptible sites for secondary solid tumors, primarily oral squamous cell carcinoma (OSCC) [6]. Several potential factors associated with secondary solid cancers are reported in HSCT patients, including the history of graft-vs-host disease (GVHD), previous long-term immunosuppressive therapy ≥24 months, use of mismatch-related donor, male gender, viral infections, genetic susceptibility, time after HSCT, age at transplant, total body irradiation (TBI) based condition, radiation exposure, immune dysfunction, hematopoietic growth factors to promote myeloid engraftment and poor Karnofsky performance status (<90) at HSCT [2].

Among the known risk factors, TBI-based condition or radiation exposure may be the single most important risk factor for the development of solid malignancies. The severity of chronic GHVD and the use of immunosuppressive therapy have also been shown to significantly contribute to excess risk [2]. 

Furthermore, the risk of specific types of solid malignancies seems to be related to exposure to different risk factors. For example, one of the largest international studies on the onset of solid cancers after HSCT demonstrated that the risk of developing a non–squamous cell carcinoma (non-SCC) following conditioning radiation was highly dependent on age at exposure. Among patients irradiated at ages under 30 years, the relative risk of non-SCC was nine times that of nonirradiated patients, while the comparable risk for older patients was 1.1 [9]. TBI-based condition is associated with a several-fold higher risk of cutaneous melanoma, thyroid cancer, breast cancer, soft tissue, and brain tumors, whereas GVHD increases the risk of OSCC by approximately fivefold [2]. In addition, the use of myeloid growth factors to promote engraftment may also be associated with an increased risk of breast cancer; The risk of lung cancer is increased among older patients and in recipients with a history of smoking before HSCT [2].

GVHD is a systemic disorder that occurs when the transplanted immunocompetent donor T lymphocytes react against the recipient tissue, recognized as foreign, due to differences in minor and major histocompatibility antigens. The grade of human leukocyte antigen (HLA) mismatch is considered one of the main risk factors for GVHD onset [10].

GVHD has been classically categorized into acute (aGVHD) and chronic (cGVHD), based on the timing of onset (before and after 100 days post-transplant, respectively). A-GVHD is T-cell mediated and presents in nearly 50–70% of HSCT, with mortality rates of 27–92%, mainly involving the gastrointestinal tract, skin, and liver. The severity of aGVHD is assessed by the degree of impairment of the involved organs: grade I is considered mild; grade II as moderate; grade III as severe, and grade IV as life-threatening [10,11,12].

C-GVHD is B and T-cell-mediated and affects approximately 50% of HSCT patients, with a mortality rate of approximately 25% [10]. It is characterized by the involvement of different organs and systems, such as the gastrointestinal tract, skin, liver, lung, musculoskeletal and genitourinary systems, and oral cavity, causing a progressive worsening of the quality of life [10,12]. 

**Figure 1:**
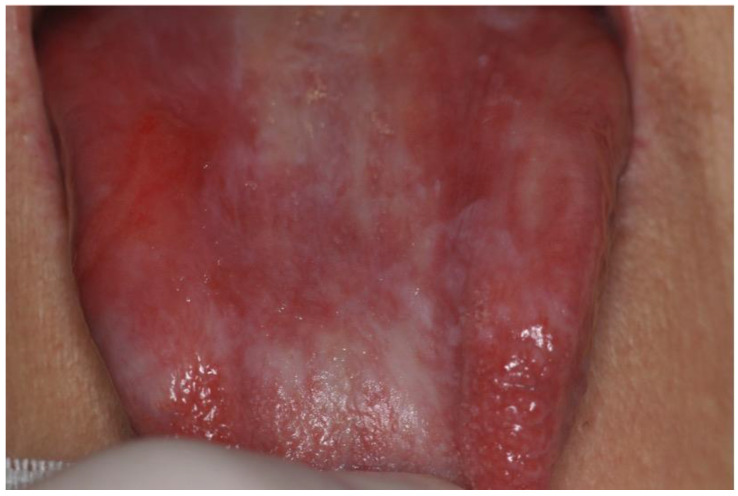
Keratotic plaque lesions on the lingual dorsum associated with severe atrophy and micro-ulcerations in a patient affected by oral cGVHD.

**Figure 2:**
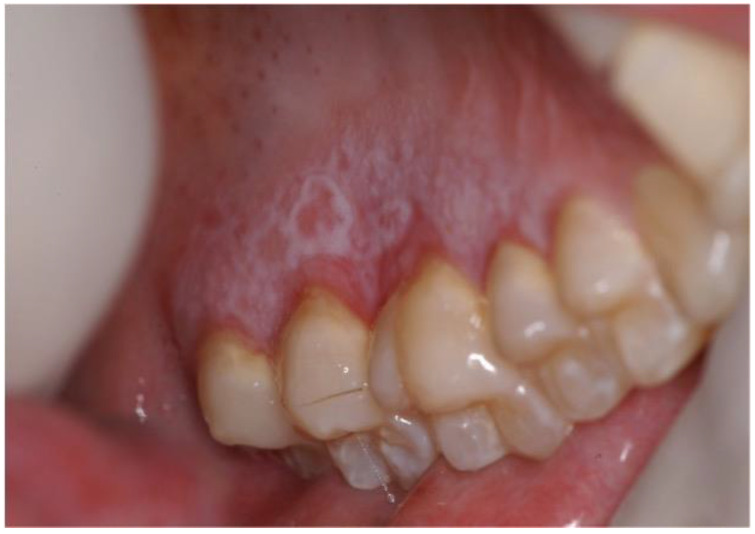
Patient affected by oral cGVHD showing keratotic lesions with papular pattern and tendency to the reticular pattern in the palatal area. There is also a paramarginal gingival erythema.

Oral cGVHD has a reported prevalence ranging from 45% to 83%, and it comprises three different patterns that can coexist: oral lichenoid lesions, mostly affecting the labial and buccal mucosa, the tongue, and the palate, manifesting as hyperkeratotic striae, patches, plaques, papules, atrophy, erosions, and ulcerations; salivary dysfunction with xerostomia; perioral fibrosis with restricted mouth-opening (Figure 1 and Figure 2) [1,10]. Furthermore, some clinical changes of oral cGVHD such as vasculitis-like features or a telangiectatic appearance of the mucosa, inflammation, and loss of the stippling of the attached gingiva may represent early alterations of oral cGVHD [13]. 

CGVHD, particularly in the case of multiorgan involvement, requires systemic corticosteroids and/or other immunomodulatory agents. However, when the oral cGVHD is resistant to systemic treatment or when the oral tissues are the only organ involved, intensive topical corticosteroid therapy is considered the mainstay of oral mucosal cGVHD management [11].

Oral cGVHD has a lead role in HSCT-related carcinogenesis, because of local immunologic dysregulation. CGVHD determines a long-term inflammation of the oral mucosa, with an upregulation of cytokines and the generation of reactive oxygen species, which are thought to be risk factors for the onset of dysplastic lesions. Furthermore, the immunologically mediated chronic injury during cGVHD may predispose to genomic instability and to oncological transformation [10].

Evidence from the current literature shows that the majority of HSCT patients, who have been developed an OSCC, suffered from the oral form of cGVHD before the diagnosis of oral malignancy [12]; furthermore, the development of solid malignancies seems to occur at anatomic sites initially involved with cGVHD-related inflammatory processes [14].

OSCC generally occurs with a latency period of 1.0 to 20.8 years after HSCT, which results in poor life quality and non-relapse mortality [1]. The preferential sites of onset are the tongue, buccal mucosa, and lips, with clinical phenotypes resembling other forms of OSCC with exophytic or endophytic ulcerated lesions [10].

Since the risk of developing OSCC increases with time post-transplantation, a particular attention should be paid to pediatric patients undergoing HSCT, who are likely to have long-term survival [12].

Although the risk of developing OSCC in patients with GVHD is well established, no studies report on the unique concerns regarding the population of patients who have been transplanted as children. Therefore, the purpose of the present paper was to review scientific literature to extrapolate all the published data on HSCT pediatric patients, affected by oral GVHD, who have been developed OSCC.

## 2. Materials and Methods

MEDLINE/PubMed and Web of Science literature searches were performed, using the following keywords in different combinations: “graft versus host disease” (GVHD), “oropharyngeal cancer”, “children”, “head and neck cancer”, “pediatric”, “oral squamous cell carcinoma” (OSCC), “hematopoietic stem cell transplantation” (HSCT), “oral cancer”. Studies were included if fulfilled the following eligibility requirement: to report data on patients with oral GVHD, affected by OSCC, who were of pediatric age at the time of the hematopoietic stem cell transplantation. Studies were excluded if reporting data on patients with oral GVHD, affected by OSCC, who were not of pediatric age at the time of the HSCT and when it was not specified if GVHD in patients submitted to HSCT in pediatric age involved oral cavity.

The following data were provided, when available, for each patient: type of disease requiring HSCT; age at transplantation; gender; stem cell source; HLA compatibility; type of conditioning regimen; GVHD prophylaxis; aGVHD/cGVHD (time lapse between HSCT and aGVHD/cGVHD, sites involved, aGVHD/cGVHD grading/severity, therapy); time lapse between HSCT and OSCC; SCC site; OSCC staging; OSCC treatment; histology; outcome.

## 3. Results

**Figure 3:**
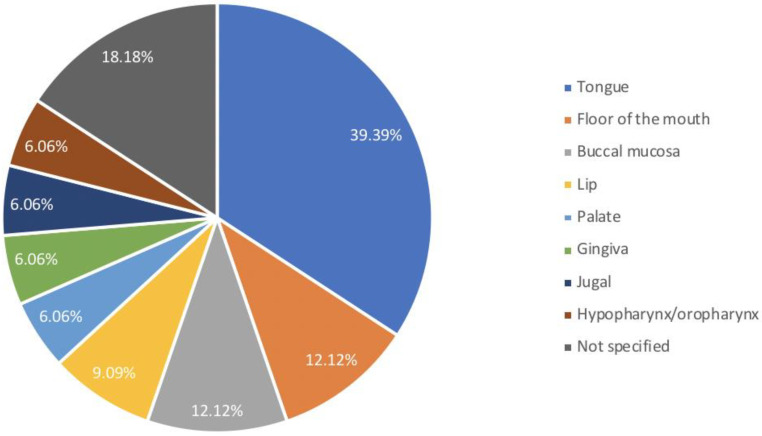
Frequency of involvement of oral sites by oropharyngeal cancer.

The electronic search yielded a total of 1256 titles that were considered potentially relevant. After removing the duplicates and reviewing the titles and abstracts, the full text of 57 articles was read. Of these, 15 studies on a total of 33 patients were included in this review. The average age of the patients at the time of HSCT was 10.3 years (4–18 years), 21 (63.64%) were men, and 12 (36.36%) were females. In all cases where the data was reported, bone marrow was the stem cell source (17 pts; 51.51%). HLA compatibility was available for 24 (72.73%) patients, and the majority of patients had a donor with adequate compatibility: this is, 14 (58.33%) RD, 5 (20.83%) HLA-id, 4 (16.67%) MUD, 1 (4.17%) MMD. The type of conditioning regimen was described in 30 cases; of these, the RIC and the MAC were used in 20 (66.67%) and 10 (33.33%) patients, respectively. Twenty patients (60.61%) developed aGVHD. No more data were available about involved sites and grading of aGVHD. All patients developed cGVHD, which, therefore, in 13 (39.39%) cases was not preceded by aGVHD. Oral mucosa was the principal involved site in all cases of cGVHD. Multiple sites involvement was reported in 15 (45.45%) patients with the following frequency: 12 (80%) other mucous membranes, 7 (46.67%) skin, 6 (40%) eyes, 3 (20%) liver, and 1 (6.67%) lungs. Data regarding oral cancer (OC) in cGVHD after HSCT showed that the mean time elapsed between HSCT and OC was 10.8 years (2–32.8). Figure 3 showed the frequency with which intraoral sites are involved in cancer.

The tongue was the most frequently involved site (13 pts; 39.39%) followed by the floor of the mouth (4 pts; 12.12%), buccal mucosa (4 pts; 12.12%), lip (3 pts; 9.09%), palate (2 pts; 6.06%), gingiva (2 pts; 6.06%), jugal (2 pts; 6.06%), hypopharynx/oropharynx (2 pts; 6.06%). In six (18.18%) cases the site of cancer was not specified. In five (15.15%) patients an involvement of two intraoral sites was reported: of these, in two cases the second oncological manifestation followed the first for 4 and 6 months, respectively. Histological examination demonstrated OSCC in 33 patients, with detection of HPV positivity in two cases. To regard the treatment of OC, the majority of the patients were treated with surgical therapy (25 pts; 75.76%). Radiotherapy and chemotherapy were used as monotherapy in three (9.09%) patients and one (3.03%) patient, respectively, and as adjuvant therapies in seven (21.21%) and two (6.06%) cases, respectively. In two (6.06%) cases only best supportive care was reported, and in one (3.03%) case there is no data about the therapy. There were 19 (57.58%) deaths occurring between 2 and 46.5 months after OC diagnosis. Eleven patients survived with a median follow-up of 34 months. All data collected are summarized in Table 1.

## 4. Discussion

OSCC is a significant cause of public health concern worldwide, representing one of the ten most common cancers in the world with an overall 5-year survival rate equal to 65% [6,28]. It mainly affects elderly males who have a long history of smoking tobacco and drinking alcohol. Other risk factors include: oral human papilloma virus (HPV) infection, ultraviolet radiation, and immune deficiency. Despite OSCC being rare among children, recent research showed that several cases of oral SCC in children and young adults were secondary cancers associated with HSCT [23].

A very recent systematic review and meta-analysis proved that patients with GVHD undergoing HSCT had a higher risk of developing OSCC compared to patients not exposed to this condition, even for a very long time after HSCT [7]. While the frequency of GVHD is usually lower in pediatric patients than in adult populations, the oral cavity can be affected by both acute and chronic forms of the disease [29]. So, young HSCT patients with GVHD, supposed to be long-term survivors, may be at high risk of developing OSCC [24].

Therefore, the present review aimed at extracting from the scientific literature all the published data on patients with oral GVHD, affected by OSCC, who were of pediatric age at the time of the transplant. Thus, the analysis of the collected data provided relevant considerations. 

Gender distribution supported the observation by Gervazio et al. that OSCC in patients with GVHD had a predilection for the male gender [7], though males were only slightly more likely than females to undergo HSCT [6].

Among the selected studies, data on HLA compatibility were available for 24 patients out of 33, of which only one male patient received bone marrow from a sex-mismatched donor. This condition is well known to be associated with a higher incidence of GVHD and inferior survival as a result of allogeneic immune responses against minor histocompatibility antigens encoded on the Y-chromosome of a male recipient (H-Y antigens) [30].

In relation to the conditioning regimen, the pre-treatment was specified in 30 out of 33 patients, of those 10 were myeloablative conditioning and 20 were reduced-intensity conditioning. The myeloablative conditioning regimens, such as total-body irradiation and/or chemotherapy, can influence the risk of secondary solid malignancies in HSCT patients [7].

Regarding chronic immunosuppressive therapy, it has been speculated that long-term immune suppression may compromise immune surveillance and exert a cocarcinogenic effect on the genetic damage caused by chemoradiation [19]. In particular, the immunosuppressive treatment with cyclosporine and azathioprine was considered a significant risk factor for secondary malignant tumors [31]. In the selected studies the type of drugs utilized for immunosuppressive therapy was specified for about one-third of patients; thus, any hypothesis concerning the correlation between specific immunosuppressive treatments and cancer onset was not possible due to lack of data.

In relation to the indication for HSCT, among the selected studies, 12 patients out of 33 were affected by Fanconi Anemia (FA), a genetically and phenotypically heterogeneous autosomal recessive, dominant or X-linked disorder, characterized by congenital abnormalities, bone marrow failure, and increased risk for cancer [32]. HSCT is considered the treatment of choice in FA patients who develop hematopoietic failure, but it may predispose to late cancer development by adding risk factors; among these, a strong association between GVHD and late malignancies has been demonstrated [20,27].

From the analysis of the reported studies emerged that none of the patients developed only aGVHD, 20 patients developed both acute and chronic forms, and 13 patients presented with cGVHD exclusively. This was in line with the study of Curtis et al., who found that, compared to patients without GVHD, the risk of solid malignancies was not elevated for patients with aGVHD; however, a previous history of aGVHD further increased the risk of solid malignancies among patients with cGVHD [33]. In relation to the GVHD characteristics, most of the papers included in the present review failed to report significant information both on clinical phenotype and exact intraoral site. Only three authors clearly described how GVHD diagnosis was made [6,22,27]. Data on histological analysis were rarely reported. Only three studies reported cGVHD clinical features, mainly described as lichenoid lesions [18,20,24]. Only in seven cases, the description of the cGVHD severity was available. The lack of this information prevents to formulate any hypothesis on the possible correlation between specific anatomical sites and a higher risk of malignant transformation; furthermore, it was also not possible to correlate cGVHD severity with an increased risk of OSCC onset. 

The time lapse between HSCT and OSCC, among the selected studies, was about 10 years. This was in accordance with other researchers, who found that the risk of solid malignancies may increase with the duration of follow-up after HSCT because of the long latency of such malignancies [2,34]. Furthermore, patients developed OSCC at a younger age if compared to that of the general population.

One of the largest international studies on this matter demonstrated that the risk of solid malignancies increased from 1.6-fold higher than the general population in the first 5 years after HSCT to 4.5-fold higher among survivors of 10 or more years [9]. According to Danylesko and Shimoni, there is no evidence for any plateau in the incidence rate; instead, the slope of the curve continues to show a steadily increased incidence with increased follow-up [35].

The pathogenesis of secondary solid malignancies after HSCT is multifactorial [2]. Therapeutic agents, such as chemotherapy and radiation, can cause breaks in double-strand DNA, resulting in gene mutations, deletions and translocations, and genomic instability conferred by loss of DNA repair [2,35]. Genomic alterations in the mucosal epithelium, as shown by microsatellite instability, have been frequently reported after HSCT, particularly in tissues affected by GVHD, and may contribute to the onset of secondary solid malignancies; immunologic alterations may also be involved in the pathogenesis [2,35]. In the context of prolonged immune suppression, pre-existing or new infections with oncogenic viruses, such as HPV and EBV, could play an etiologic role in many post-transplant solid cancers [21]. Moreover, somatic mutations in the tumor suppressor gene TP53 are frequently reported in these tumors [34]. As highlighted by Adhikari et al., these factors may cooperatively predispose HSCT recipients to an improved risk of solid malignancies [2].

Regarding HPV status, two p16/HPV-positive OSCC cases were reported [21,25].

Although HPV infection is well-recognized to be associated with head and neck squamous cell carcinoma (HNSCC), being involved in about one-quarter of the cases in the general population, the role of HPV in the development of HNSCC in post-HSCT patients has not yet been clarified [21,25]. The reports about HPV involvement in HNSCC after HSCT are quite scarce, suggesting the need for routine HPV screening in HSCT patients [21,25].

In the present review, the development of two synchronous OSCCs was described by two authors, respectively [16,24]. Montebugnoli et al. reported the development of two synchronous carcinomatous lesions on the tongue and the floor of the mouth in a young female patient 17 years after HSCT [24]. Somers et al. described the development of two synchronous carcinomatous lesions on the tongue in a female teenage patient 8 years after HSCT [16].

As stated by Abdelsayed et al., the presence of synchronous carcinomas indicated that OSCC in HSCT patients with GVHD may have an aggressive biologic potential with an increased tendency for recurrence and the development of new lesions [14].

Furthermore, secondary tumors were reported to respond less well to standard therapy compared with de novo OSCC [29]. In fact, as it is possible to deduce from the reported data, the prognosis for HSCT pediatric patients, affected by oral cGVHD, who have been developed OSCC, remained poor, despite therapeutic advances. Therefore, a strict surveillance, such as allowing an early diagnosis, continues to be the key element to increase the survival rate [31]. Patients, who are at high-risk for SCC of the oral cavity such as those with oral chronic GVHD and Fanconi’s anemia, should undergo oral evaluations every 6 months. Avoidance of smoking and alcohol may also reduce the risk of oral cancer [2].

The present review had certain limitations that should be addressed. First, from the literature search it was not always possible to quantify how many patients with oral GVHD, who underwent HSCT as children, developed OSCC, because, in some large surveys, data on age at HSCT were reported exclusively as median age. Furthermore, other studies failed to specify if GVHD in patients submitted to HSCT in pediatric age involved oral cavities. Another limitation concerned possible under-reporting by transplant centers to the registry as transplant centers may not have information on long-term HSCT survivors, which is important when considering secondary cancer screening in patients who underwent HSCT as children.

## 5. Conclusions

Children who undergo HSCT will continue to be at increased risk for the development of secondary solid cancers over the course of their lifetime and lifelong surveillance is suggested to reduce post-transplant mortality due to secondary tumors. A careful oral examination performed by an oral medicine specialist every 6 months is recommended for patients that have developed cGVHD, looking at modifications in clinical morphology, evaluating nonhomogeneous patterns, and treating suspicious lesions with incisional biopsy to rule out the presence of dysplasia and local malignancies. Additionally, a photo-documentation should be performed regularly. Furthermore, patients should be informed about the risks and symptoms of OSCC, should perform frequent self-inspection of the oral cavity and report any suspicious lesions, and should be encouraged to avoid high-risk behaviors.

Finally, the role of dentists or oral surgeons should not be underestimated and increasing awareness of the association between oral GvHD and OSCC can help to make a prompt diagnosis, allowing a more effective clinical management, and limiting morbidity and mortality in long-term survivors of HSCT patients.

## Figures and Tables

**Table 1:** Published data about oral cancer among pediatric patients affected by GVHD after HSCT.

Authors/Year of Publication/ Study Type	Disease	Age at Trasplantation/ Gender	Stem Cell Source	HLA Compatibility	Type of Conditioning Regimen	Conditioning Regimen	GVHD Prophylaxis	aGVHD	Time Lapse between HSCT and aGVHD (Months)	Sites Involved aGVHD	Grading aGVHD	Therapy aGVHD	cGVHD	Time Lapse between HSCT and cGVHD (Months)	Sites Involved cGVHD	Grading cGVHD	Therapy cGVHD	Time Lapse between HSCT and OSCC (Year)	Cancer Site	Staging (TNM)	Histology	Treatment	Outcome
Socié et al., 1991 [15] Retrospective study	SAA	12/M	-	HLA-id	RIC	CY, TAI	MTX	No	N/A	N/A	N/A	N/A	Yes	-	Oral cavity	Extensive	-	7.9	Lip	-	SCC	SE, RT	-
Somers et al., 1995 [16] Case report	FA	8/M	-	-	RIC	CY, TAI	CS, S	Yes	-	-	-	-	Yes	60	Oral cavity	-	AZA	8	Tongue	-	SCC	SE	-
																			Tongue (after 6 months)	-		SE	
Millen et al., 1997 [17] Case report	FA	8/F	BM	HLA-id	MAC	LD CY, TBI	-	Yes	-	Skin, Gut	III	-	Yes	-	Skin, Liver, Oral cavity	-	CS, AZA	10	Buccal mucosa	-	SCC	SE, ND, RT	Dead after 3 months
Otsubo et al., 1997 [18] Case report	SAA	16/F	-	HLA-id	RIC	CY, TLI	CS, MTX	Yes	1	forearms	II	S	Yes	4	Oral cavity	-	CS, S	4	Gingiva	T3N0M0	SCC	SE, ND	Alive (no information on follow-up)
Shimada et al., 2005 [19] Retrospective study	NHL	17.5 M	BM	HLA-id	MAC	E, MEL, TBI	-	Yes	-	-	-	-	Yes	-	Oral cavity	Mild	-	11.5	Oral cavity	-	SCC		Dead after 3 months
Salum et al., 2006 [20] Case report	FA	5/M	BM	-	RIC	CY	CS, MTX	No	N/A	N/A	N/A	N/A	Yes	10	Buccal mucosa	-	SC	11	Tongue	T3N0M0	SCC	CH, RT	Dead after 4 months
														84	Tongue	-	No treatment						
														94	Palate, Gingiva, Upper lip, Buccal mucosa	-	LC						
Byun et al., 2008 [21] Case report	CML	12/F	BM	-	MAC	CY, BU	CS, S	No	N/A	N/A	N/A	N/A	Yes	6	Oral cavity, Skin, Liver, Eyes, Lungs	-	CS, S	5	Tongue	T2N0M0	SCC*	SE, ND	Alive (follow-up: 5 months)
Masserot et al., 2008 [22] Case series	FA	11.7/M	BM	MUD	MAC	CY LD, TBI, ATG	Yes (N/S)	No	N/A	N/A	N/A	N/A	Yes	-	Mucosal, Skin	-	-	10	Tongue, Palate	T1N0M0	SCC	SE, ND	Alive (follow-up:9 months)
	FA	11.2/M	BM	RD	RIC	CY LD, TAI	Yes (N/S)	Yes	-	-	III	S, CS, ATG	Yes	-	Mucosal	-	-	5.7	Tongue	T2N+M0	SCC	SE, RT	Dead after 5.5 months
	FA	9.7/M	BM	RD	RIC	CY LD, TAI	Yes (N/S)	Yes	-	-	III	S, CS, ATG	Yes	-	Mucosal	-	-	7.8	Hypopharynxs	T4N2cMx	SCC	CH	Dead after 6 months
	FA	8.9/F	BM	MUD	MAC	CY LD, TAI, ATG	Yes (N/S)	Yes	-	-	II	S, CS	Yes	-	Mucosal	-	-	8.3	Tongue	T1NxMx	SCC	SE	Dead after 6 months
	FA	5.2/M	BM	RD	RIC	CY LD, TAI	Yes (N/S)	Yes	-	-	III	S, CS	Yes	-	Mucosal, Skin	-	-	15.3	Floor of the mouth	T1N0M0	SCC	SE, ND	Alive (follow-up: 23 months)
	FA	7.3/M	BM	RD	RIC	CY LD, TAI	Yes (N/S)	Yes	-	-	II	S, CS	Yes	-	Mucosal, Eye	-	-	12.4	Oropharynx	T4N2cMx	SCC	RT	Dead after 4.5 months
	FA	11.2/M	BM	RD	RIC	CY LD, TAI	Yes (N/S)	Yes	-	-	II	S, CS	Yes	-	Mucosal	-	-	7	Floor of the mouth	T3N0Mx	SCC	SE, ND	Dead after 16 months
	FA	4.6//M	BM	RD	RIC	CY LD, TAI	Yes (N/S)	Yes	-	-	IV	S, CS, ATG	Yes	-	Mucosal, Skin, Eye, Liver	-	-	5.5	Tongue	T3N0Mx	SCC	RT	Dead after 2.5 months
	FA	6.5/F	BM	RD	MAC	CY HD, TBI	Yes (N/S)	Yes	-	-	III	S, ATG	Yes	-	Mucosal, Eye	-	-	21.6	Jugal, Floor of the mouth	T1NxMx	SCC	SE, CRY	Dead after 46.5 months
	FA	10.3/M	BM	RD	RIC	CY LD, TAI	Yes (N/S)	Yes	-	-	II	S, CS	Yes	-	Mucosal	-	-	14	Palate, Jugal	T1NxMx	SCC	SE	Dead after 11 months
	FA	14.3/F	BM	RD	RIC	CY LD, TAI	Yes (N/S)	Yes	-	-	II	S, CS	Yes	-	Mucosal, Skin, Eye	-	-	9.4	Gingiva	T2N0Mx	SCC	SE, ND	Dead after 6.5 months
	FA	7.5/F	BM	MUD	RIC	CY LD, TAI	Yes (N/S)	Yes	-	-	II	S, CS	Yes	-	Mucosal, Eye		-	19.2	Tongue	T1N0M0	SCC	SE, ND	Dead after 41 months
Tomihara et al., 2009 [23] Case report	ALL	11/M	BM	MMD	MAC	TBI	-	No	N/A	N/A	N/A	N/A	Yes	-	Oral cavity	-	-	13	Buccal mucosa		SCC	RT, ND	Alive (no information on follow-up)
Montebugnoli et al., 2011 [24] Case report	TM	9/F	-	-	-	-	CS, S	No	N/A	N/A	N/A	N/A	Yes	6	Buccal mucosa	-	LC, AMD	17	Tongue	T3N0M0	SCC	SE, ND	Alive (follow-up: 2 years)
																			Floor of the mouth (after 4 months)	T2N0M0		SE	
Katz et al., 2014 [25] Case report	AML	18/M	-	MUD	-	-	-	No	N/A	N/A	N/A	N/A	Yes	-	Lower lip	-	-	9	Upper lip	T1N0M0	SCC*	RT	Alive (no information on follow-up)
Torres-Pereira et al., 2014 [26] Case report	FA	8/F	-	HLA-id	RIC	CY	CS, MTX	No	N/A	N/A	N/A	N/A	Yes	-	Buccal mucosa	Mild	No treatment	10	Tongue	-	SCC	SE, ND	Alive (follow-up: 5 years)
Bonfim et al., 2016 [27] Retrospective study	FA	4/M	-	RD	RIC	CY	Yes (N/S)	No	N/A	N/A	N/A	N/A	Yes	-	Oral cavity	-	-	11	Oral cavity	T3N0M0	SCC	BSC	Dead
	FA	6/M	-	RD	RIC	CY	Yes (N/S)	No	N/A	N/A	N/A	N/A	Yes	-	Oral cavity	-	-	5	Oral cavity	T2NxM0	SCC	SE	Dead
	FA	6/F	-	RD	RIC	CY	Yes (N/S)	Yes	-	-	-	.	Yes	-	Oral cavity	-	-	6	Oral cavity	T2N0M0	SCC	SE, RT	Dead
	FA	7/M	-	RD	RIC	CY	Yes (N/S)	Yes	-	-	-	.	Yes	-	Oral cavity	-	-	8	Oral cavity	T3NxM0	SCC	BSC	Dead
	FA	10/M	-	RD	RIC	CY	Yes (N/S)	Yes	-	-	-	.	Yes	-	Oral cavity	-	-	5	Oral cavity	T1N0M0	SCC	SE	Alive (follow-up: 5 years)
Liu et al., 2020 [1] Case report	AML	14/F	-	-	-	-	CS	No	N/A	N/A	N/A	N/A	Yes	-	Oral cavity, Skin	-	CS, MTX	2	Buccal mucosa	TisN0M0	SCC	SE	Alive (follow-up:4 years)
Santarone et al., 2021 [6] Retrospective study	SAA	15/M	-	-	RIC	CY	Yes (N/S)	Yes	-	-	III	Yes (N/S)	Yes	-	Oral Cavity	Extensive	CS, S, AZA	32.8	Lower lip	T1NxM0	SCC	SE, RT	Dead after 14 months
	TM	14/M	-	-	MAC	BU CY	Yes (N/S)	Yes	-	-	I	Yes (N/S)	Yes	-	Oral Cavity	Extensive	CS, S	11.8	Tongue	T2NxM0	SCC	SE, CH	Dead after 2 years
	TM	13M	-	-	MAC	BU CY	Yes (N/S)	No	N/A	N/A	N/A	N/A	Yes	-	Oral Cavity	Extensive	CS, S	21.1	Tongue	T3N0M0	SCC	SE, CH, RT	Alive (follow-up:4 years)
	ALL	18/F	-	-	MAC	TBI TH FLU	Yes (N/S)	No	N/A	N/A	N/A	N/A	Yes	-	Oral Cavity	Extensive	CS, S, ECP	9.8	Buccal mucosa	T2N0M0	SCC	SE	Alive (follow-up: 2.5 years)

SAA: severe aplastic anemia; HLA-id: HLA identical; RIC: reduced intensity conditioning; CY: cyclophosphamide; TAI: thoracoabdominal irradiation; MTX: methotrexate; N/A: Not applicable; SE: surgical excision; RT: radiotherapy; FA: Fanconi Anemia; CS: cyclosporine; S: steroids; AZA: azathioprine; SCC: squamous cell carcinoma; BM: bone marrow; MAC: myeloablative conditioning; LD: low dose; TBI: total body irradiation; ND: neck dissection; TLI: total lymphoid irradiation; NHL: non-Hodgkin’s lymphoma; E: etoposide; Mel: melphalan; SC: systemic corticosteroids; LC: local corticosteroids; CH: chemotherapy; CML: chronic myeloid leukaemia; BU: busulfan; ALL: acute lymphoblastic leukemia; MMD: mismatched donor; TM: thalassemia major; AMD: antimycotic drugs; AML: acute myeloid leukaemia; MUD: matched unrelated donor; N/S: Not specified; RD: related donor; BSC: best supportive care; ATG: antithymocyte globulin; HD: high dose; CRY: cryotherapy; TH: thiotepa; FLU: fludarabine; ECP: extracorporeal photopheresis. *p16/HPV-positive OSCC.

## Data Availability

The data that support the findings of this study are available from the corresponding author upon reasonable request.

## References

[1] Liu Y., Yuan W., Li M., Cheng L., Yang J., Yin B., Huang X. (2020). In situ buccal carcinoma in a teenager after hematopoietic stem cell transplantation: A case report. Medicine.

[2] Adhikari J., Sharma P., Bhatt V.R. (2015). Risk of secondary solid malignancies after allogeneic hematopoietic stem cell transplantation and preventive strategies. Future Oncol. (Lond. Engl.).

[3] Bosi A., Bartolozzi B. (2010). Safety of bone marrow stem cell donation: A review. Transplant. Proc..

[4] Walter R.B., Pagel J.M., Gooley T.A., Petersdorf E.W., Sorror M.L., Woolfrey A.E., Hansen J.A., Salter A.I., Lansverk E., Stewart F.M. (2010). Comparison of matched unrelated and matched related donor myeloablative hematopoietic cell transplantation for adults with acute myeloid leukemia in first remission. Leukemia.

[5] Bhatia S. (2011). Long-term health impacts of hematopoietic stem cell transplantation inform recommendations for follow-up. Expert Rev. Hematol..

[6] Santarone S., Natale A., Angelini S., Papalinetti G., Vaddinelli D., Di Bartolomeo A., Di Bartolomeo P. (2021). Secondary oral cancer following hematopoietic cell transplantation. Bone Marrow Transplant..

[7] Gervazio T.C., Silva J.K., Evangelista K., Cavalcanti M., Silva M., Yamamoto-Silva F.P., Silva B. (2022). Risk of oral cancer in patients with graft-vs-host disease: A systematic review and meta-analysis. Oral Surg. Oral Med. Oral Pathol. Oral Radiol..

[8] Demarosi F., Soligo D., Lodi G., Moneghini L., Sardella A., Carrassi A. (2005). Squamous cell carcinoma of the oral cavity associated with graft versus host disease: Report of a case and review of the literature. Oral Surg. Oral Med. Oral Pathol. Oral Radiol. Endod..

[9] Rizzo J.D., Curtis R.E., Socié G., Sobocinski K.A., Gilbert E., Landgren O., Travis L.B., Travis W.D., Flowers M.E., Friedman D.L. (2009). Solid cancers after allogeneic hematopoietic cell transplantation. Blood.

[10] Leuci S., Coppola N., Blasi A., Ruoppo E., Bizzoca M.E., Lo Muzio L., Marano L., Risitano A.M., Mignogna M.D. (2020). Oral Dysplastic Complications after HSCT: Single Case Series of Multidisciplinary Evaluation of 80 Patients. Life.

[11] Elad S., Aljitawi O., Zadik Y. (2021). Oral Graft-Versus-Host Disease: A Pictorial Review and a Guide for Dental Practitioners. Int. Dent. J..

[12] Janowiak-Majeranowska A., Osowski J., Mikaszewski B., Majeranowski A. (2022). Secondary Oral Cancer after Systemic Treatment of Hematological Malignancies and Oral GVHD: A Systematic Review. Cancers.

[13] Gomes A.O., Torres S.R., Maiolino A., Dos Santos C.W., Silva Junior A., Correa M.E., Moreira M.C., Gonçalves L. (2014). Early and late oral features of chronic graft-versus-host disease. Rev. Bras. Hematol. Hemoter..

[14] Abdelsayed R.A., Sumner T., Allen C.M., Treadway A., Ness G.M., Penza S.L. (2002). Oral precancerous and malignant lesions associated with graft-versus-host disease: Report of 2 cases. Oral Surg. Oral Med. Oral Pathol. Oral Radiol. Endod..

[15] Socié G., Henry-Amar M., Cosset J.M., Devergie A., Girinsky T., Gluckman E. (1991). Increased incidence of solid malignant tumors after bone marrow transplantation for severe aplastic anemia. Blood.

[16] Somers G.R., Tabrizi S.N., Tiedemann K., Chow C.W., Garland S.M., Venter D.J. (1995). Squamous cell carcinoma of the tongue in a child with Fanconi anemia: A case report and review of the literature. Pediatr. Pathol. Lab. Med. J. Soc. Pediatr. Pathol. Affil. Int. Paediatr. Pathol. Assoc..

[17] Millen F.J., Rainey M.G., Hows J.M., Burton P.A., Irvine G.H., Swirsky D. (1997). Oral squamous cell carcinoma after allogeneic bone marrow transplantation for Fanconi anaemia. Br. J. Haematol..

[18] Otsubo H., Yokoe H., Miya T., Atsuta F., Miura N., Tanzawa H., Sato K. (1997). Gingival squamous cell carcinoma in a patient with chronic graft-versus-host disease. Oral Surg. Oral Med. Oral Pathol. Oral Radiol. Endod..

[19] Shimada K., Yokozawa T., Atsuta Y., Kohno A., Maruyama F., Yano K., Taji H., Kitaori K., Goto S., Iida H. (2005). Solid tumors after hematopoietic stem cell transplantation in Japan: Incidence, risk factors and prognosis. Bone Marrow Transplant..

[20] Salum F.G., Martins G.B., de Figueiredo M.A., Cherubini K., Yurgel L.S., Torres-Pereira C. (2006). Squamous cell carcinoma of the tongue after bone marrow transplantation in a patient with Fanconi anemia. Braz. Dent. J..

[21] Byun J.H., Park B.W., Kim J.R., Lee G.W., Lee J.H. (2008). Squamous cell carcinoma of the tongue after bone marrow transplant and graft-versus-host disease: A case report and review of the literature. J. Oral Maxillofac. Surg. Off. J. Am. Assoc. Oral Maxillofac. Surg..

[22] Masserot C., Peffault de Latour R., Rocha V., Leblanc T., Rigolet A., Pascal F., Janin A., Soulier J., Gluckman E., Socié G. (2008). Head and neck squamous cell carcinoma in 13 patients with Fanconi anemia after hematopoietic stem cell transplantation. Cancer.

[23] Tomihara K., Dehari H., Yamaguchi A., Abe M., Miyazaki A., Nakamori K., Hareyama M., Hiratsuka H. (2009). Squamous cell carcinoma of the buccal mucosa in a young adult with history of allogeneic bone marrow transplantation for childhood acute leukemia. Head Neck.

[24] Montebugnoli L., Gissi D.B., Marchetti C., Foschini M.P. (2011). Multiple squamous cell carcinomas of the oral cavity in a young patient with graft-versus-host disease following allogenic bone marrow transplantation. Int. J. Oral Maxillofac. Surg..

[25] Katz J., Islam M.N., Bhattacharyya I., Sandow P., Moreb J.S. (2014). Oral squamous cell carcinoma positive for p16/human papilloma virus in post allogeneic stem cell transplantation: 2 cases and review of the literature. Oral Surg. Oral Med. Oral Pathol. Oral Radiol..

[26] Torres-Pereira C.C., Stramandinoli-Zanicotti R.T., Amenábar J.M., Sassi L.M., Galbiatti Pedruzzi P.A., Piazzetta C.M., Bonfim C. (2014). Oral squamous cell carcinoma in two siblings with Fanconi anemia after allogeneic bone marrow transplantation. Spec. Care Dent. Off. Publ. Am. Assoc. Hosp. Dent. Acad. Dent. Handicap. Am. Soc. Geriatr. Dent..

[27] Bonfim C., Ribeiro L., Nichele S., Bitencourt M., Loth G., Koliski A., Funke V., Pilonetto D.V., Pereira N.F., Flowers M. (2016). Long-term Survival, Organ Function, and Malignancy after Hematopoietic Stem Cell Transplantation for Fanconi Anemia. Biol. Blood Marrow Transplant. J. Am. Soc. Blood Marrow Transplant..

[28] Cancer. https://www.cancer.net/cancer-types/oral-and-oropharyngeal-cancer/statistics.

[29] Majorana A., Schubert M.M., Porta F., Ugazio A.G., Sapelli P.L. (2000). Oral complications of pediatric hematopoietic cell transplantation: Diagnosis and management. Support. Care Cancer Off. J. Multinatl. Assoc. Support. Care Cancer.

[30] Nakasone H., Remberger M., Tian L., Brodin P., Sahaf B., Wu F., Mattsson J., Lowsky R., Negrin R., Miklos D.B. (2015). Risks and benefits of sex-mismatched hematopoietic cell transplantation differ according to conditioning strategy. Haematologica.

[31] Kruse A.L., Grätz K.W. (2009). Oral carcinoma after hematopoietic stem cell transplantation—A new classification based on a literature review over 30 years. Head Neck Oncol..

[32] Anak S., Yalman N., Bilgen H., Sepet E., Deviren A., Gürtekin B., Tunca F., Başaran B. (2020). Squamous cell carcinoma development in Fanconi anemia patients who underwent hematopoietic stem cell transplantation. Pediatr. Transplant..

[33] Curtis R.E., Metayer C., Rizzo J.D., Socié G., Sobocinski K.A., Flowers M.E., Travis W.D., Travis L.B., Horowitz M.M., Deeg H.J. (2005). Impact of chronic GVHD therapy on the development of squamous-cell cancers after hematopoietic stem-cell transplantation: An international case-control study. Blood.

[34] Leisenring W., Friedman D.L., Flowers M.E., Schwartz J.L., Deeg H.J. (2006). Nonmelanoma skin and mucosal cancers after hematopoietic cell transplantation. J. Clin. Oncol. Off. J. Am. Soc. Clin. Oncol..

[35] Danylesko I., Shimoni A. (2018). Second Malignancies after Hematopoietic Stem Cell Transplantation. Curr. Treat. Options Oncol..

